# Is it possible to not perform salivary gland biopsy in targeted patients according to unstimulated salivary flow results in patients with suspected Sjögren's syndrome?

**DOI:** 10.1007/s00296-021-04840-4

**Published:** 2021-04-12

**Authors:** Agata Sebastian, Patryk Woytala, Marta Madej, Krzysztof Proc, Katarzyna Czesak-Woytala, Maciej Sebastian, Krzysztof Zub, Piotr Wiland

**Affiliations:** 1 Department of Rheumatology and Internal Medicine, Medical University of Wroclaw, Borowska 213, 50-556 Wroclaw, Poland; 2 Department of Mathematics and Cybernetics, Wroclaw University of Economics, Wroclaw, Poland; 3 Department of General, Minimally Invasive and Endocrine Surgery, Medical University of Wroclaw, Wroclaw, Poland; 4 Department and Clinic of Otolaryngology-Head and Neck Surgery, Wroclaw Medical University, Wroclaw, Poland

**Keywords:** Primary Sjögren's syndrome, Hyposalivation, Unstimulated salivary flow, Focus score, Xerostomia

## Abstract

**Introduction/objective:**

Xerostomia is one of the main symptoms of primary Sjögren's syndrome (pSS). The unstimulated salivary flow (UWS) test is one of the objective Sjögren's syndrome classification criteria used to assess xerostomia's severity. The study’s objective was to evaluate UWS rate measurements (with a threshold rate of 0.1 mL/min) in the screening of patients suspected with pSS, presenting with xerostomia in whom labial salivary gland biopsy (LSGB) should be performed. We will try to answer whether it is possible not to perform LSGB in targeted patients according to UWS results? We analyze the correlation between UWS value and focus score (FS) and anti-SSA antibodies.

**Methods:**

The study group consisted of subjects above 18 years of age with a subjective feeling of oral dryness.

**Results:**

A total of 105 subjects were qualified for the study. The final diagnosis of pSS was made in 44 patients according to the classification criteria from 2016. No age differences were identified between pSS patients and control group subjects (patients with dry mouth without autoimmune background). UWS rates were significantly lower in pSS patients than in the control group. No association was identified between UWS and focus score (FS) ≥ 1 in LSGB. No differences were observed between anti-SSA-positive and anti-SSA-negative patients in terms of age, UWS rates, FS.

**Conclusion:**

LSGB should be performed in all suspected pSS cases regardless of the UWS rate value, particularly in subjects without specific anti-SSA antibodies. In patients with suspected pSS, only less than one-half of the UWS measurements are below the value of 0.1 mL/min adopted as the threshold in the classification criteria for pSS.

## Introduction

Xerostomia is one of the main symptoms of Sjögren's syndrome (SS). Xerostomia is the subjective sensation of dry mouth, which is often, but not always, associated with hypofunction of the salivary glands. In 2016, it was included among the preliminary SS diagnostic criteria [1]. The unstimulated salivary flow (UWS) test is one of the objective SS classification criteria used to assess the severity of salivary glands’ hypofunction. A salivary flow rate of ≤ 0.1 mL/min is considered an abnormal result typical for SS. This value was first specified in the classification criteria established more than twenty years ago [2] and has not been changed to date. Recently, an increasing number of studies is being published in which the authors prove that UWS values are age- and gender-dependent [3, 4]. Unfortunately, in the majority of the cases, this test is only applied when the patient does not have a dry eye by objective measures [1].

Labial salivary gland biopsy (LSGB) is considered one of the most objective tests used to confirm the diagnosis of SS [1, 2]. Despite its diagnostic value, LSGB [5] has certain limitations [6] manifested, e.g., by false-positive results in older adults. Hence, the question naturally arises as to which patients should be subjected to LSGB with a high likelihood of SS diagnosis.

The study's objective was to evaluate UWS rate measurements in screening patients with suspected Sjögeren syndrome and presenting with xerostomia in whom LSGB should be performed. We analyze the correlation between UWS value and focus score (FS) and anti-SSA antibodies. We will try to answer whether it is possible not to perform LSGB in targeted patients according to UWS results > 0.1 mL/min? Is there a UWS value at which we can determine with high probability that we will get a focus score above 1?

## Materials and methods

The study group consisted of subjects above 18 years of age who had reported to the Rheumatology Clinic with suspected SS and subjective feeling of oral dryness between December 2018 and September 2020. Each subject had provided at least one positive response in the oral dryness symptoms inventory per the AECG/ACR criteria [1, 2] as inclusion criteria. Each person had provided written consent to undergo study procedures including UWS measurements [7], LSGB, collection of blood samples with the determination of ANA (IF), ENA (ELISA), anti-dsDNA (ELISA) antibodies, ultrasound (US) of large salivary glands (the MyLab25Gold (Esaote) with a linear head; frequency range: 12–18 MHz), and Schirmer's test. The US examination was blinded for laboratory parameters. The diagnosis of SS was confirmed based on the ACR/EULAR 2016 classification criteria [1]. Exclusion criteria included: age < 18 years, diagnosis of other autoimmune diseases, active HCV infection, sarcoidosis, IgG4-related disease, lymphoma, amyloidosis, graft-versus-host disease.

The control group consisted of patients with xerostomia with suspected pSS, who were not confirmed as having pSS or other autoimmune diseases.

The prospective-observational study was approved by the Ethics Committee of Medical University (decision number 599/2018) and carried out according to the Declaration of Helsinki.

UWS tests were carried out in the morning (8:00 am–12:00 pm). Each subject had been asked not to use any parasympathomimetic drugs during the preceding 12 h, not to smoke or drink coffee, not to use any drugs 3 h before the test, not to use any saliva substitutes 3 h before the test, as well as not to eat, drink water, and brush or floss their teeth one hour before the test.

After the test, LSGB was collected from each subject, following topical mucosal anesthesia using 10% lidocaine, for histopathological screening for lymphocytic infiltrations typical for SS as per current guidelines (focal lymphocytic sialadenitis and focus score (FS) of ≥ 1 foci/4 mm^2^) [8]. All subjects were then subjected to the ultrasound of major salivary glands to identify hypoechogenic areas and blurred and patchy salivary gland structures typical of SS as defined in the classification system proposed by De Vita [9].

### Statistical analysis

At the first stage, homogeneity between pSS and the control group was analyzed. At the second stage, patients with pSS were divided into subgroups of patients presenting with SSA antibodies (SS-A +) and patients without SSA antibodies (SS-A −). Differences between the groups were evaluated.

Statistical analyses were carried out using the Statistica 10 software package.

Mann–Whitney *U* test was used to compare the distributions of quantitative variables in two independent groups. A chi-squared test was used to verify the relationships between variables expressed using dichotomous scales. Spearman's coefficient and the corresponding statistical test were used to assess the significance of correlations between quantitative variables. The significance level was set to 0.05.

## Results

A total of 105 subjects with subjective sensation on the dry mouth (xerostomia) were qualified for the study. The final diagnosis of pSS was made in 44 patients. In 61 people with subjective xerostomia, the pSS or other defined disease was not diagnosed (control group) (Table 1).

**Table 1:** Characteristics of the pSS patients and the control group

	pSS (*n* 44)	Control group (*n* 61) with subjective xerostomia	*p* value
Patient age (years)			
Mean (SD) Range	55 (14.6) 26–81	55 (11.6) 26–75	
Sex			
Females (*n*) Males (*n*)	39 5	57 4	
Mean USF (mL/min)	0.33	0.67	.003 (test *U*)
UWS ≤ 0.1 mL/min			
*n* %	15 34	6 10	.01 (test $\chi^{2}$)
Mean FS (LSGB)	2.0	0.05	< .005 (test *U*)
FS ≥ 1 (LSGB)			
*n* %	25 57	1 1	< .05(test $\chi^{2}$)
Changes in salivary gland US scans			
Parotid			
*n* %	28 63	5 8	< .005 (test $\chi^{2}$)
Submandibular			
*n*	31	7	< .005 (test $\chi^{2}$)
%	70	11	
ANA ≥ 1:320			
*n* %	39 89	19 31	.002 (test $\chi^{2}$)

*ANA* antinuclear antibodies; *FS* focus score; *UWS* unstimulated salivary flow; *US scans* ultrasonography based on de Vita score; *LSGB* labial salivary gland biopsy; *n* number of patients

**Fig. 1:**
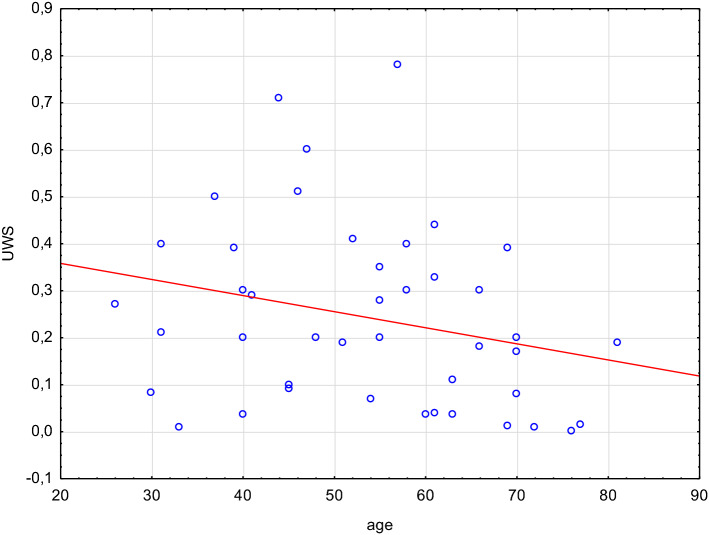
Correlation between unstimulated whole salivary flow (UWS) rate and age of patients in group with primary Sjögren syndrome

No age differences were identified between pSS and control group subjects. The majority of patients were females around 55 years of age. In the first stage, we analyzed the UWS values' dependence on patients' age (Fig. 1). Statistical analysis showed that UWS values were not significantly lower in older individuals with pSS. The calculated Spearman's correlation coefficient was − 0.28. The *p* value for the correlation significance test was − 0.23.

The Schirmer test (< 5 mm in 5 min at least in one eye) was positive in 86% (*n* = 38) of patients with pSS. The dry eye in Schirmer test (result < 15 mm in 5 min at least in one eye) was confirmed in 49% of patients (*n* = 30) from the control group, but only in 8% (*n* = 5) the Schirmer test was lower than 5 mm in 5 min.

**Fig. 2:**
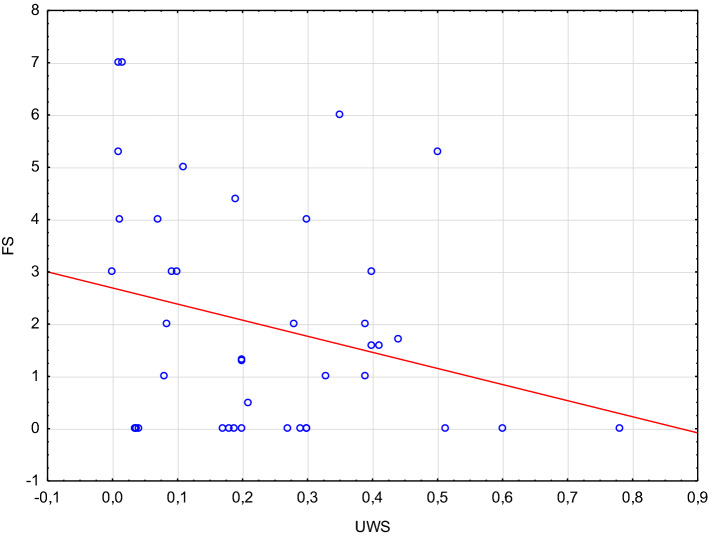
Relationship between focus score ≥ 1 (FS) and unstimulated whole salivary flow (UWS) values in patients with primary Sjögren’s syndrome

UWS rates were significantly lower in pSS patients compared to the control group of subjects with subjective xerostomia (0.33 mL/min vs 0.67 mL/min, *p* < 0.05). Likewise, lymphocytic infiltrations of salivary glands were significantly more common in pSS patients (57% vs 1%, *p* < 005). However, no association was identified between UWS and FS ≥ 1 (Fig. 2). As much as 65% of pSS patients presented with UWS of > 0.1 mL/min.

Changes in ultrasound scans of large salivary glands consisting of structural heterogeneity and hypoechogenic areas were observed more frequently in pSS patients than control subjects. Within the pSS group, changes were more frequent in submandibular salivary glands than parotid salivary glands (70% vs 63%), albeit the difference was not statistically significant (*p* > 0.05). Changes in ultrasound scans of large salivary glands were observed in 11% of control group subjects, mainly within the submandibular salivary glands. ANA at titers of ≥ 1:320 was confirmed in 31% of control group subjects. However, no criteria of SS or other systemic connective tissue disease were met by these subjects. Most frequently, antibodies observed in this group included anti-DFS70 (80%), AMA, anti-centromere and anti-Ro52 antibodies.

No differences were observed between anti-SSA + and anti-SSA − patients in terms of age, UWS rates, and presence of ultrasound imaging changes (Table 2).

**Table 2:** Characteristics of pSS patients with and without anti-SSA antibodies

	pSS-positive anti SSA ab (*n* 34)	pSS-negative anti SSA ab (*n* 10)	*p* value
Patient age (years)			
Mean (SD)	53 (14.5)	60 (14.0)	.12 (test *U*)
Mean UWS (mL/min)	0.4	0.09	.60 (test *U*)
UWS ≤ 0.1 mL/min			
*n* %	11 32	4 40	.75 (test $\chi^{2}$)
Mean FS (LSGB)	1.7	2.9	.015 (test *U*)
FS ≥ 1 (LSGB)			
*n* %	26 76	10 100	.60 (test $\chi^{2}$)
Changes in salivary gland US scans			
Parotid			
*n*	25	4	.34 (test $\chi^{2}$)
%	73	40	
Submandibular			
* n*	25	6	.72 (test $\chi^{2}$)
%	73	60	

*ANA* antinuclear antibodies; *FS* focus score; *UWS* unstimulated salivary flow; *US scans* ultrasonography based on de Vita score; *LSGB* labial salivary gland biopsy; *n* number of patients; *ab* antibodies

## Discussion

Our study results indicate that UWS rates are significantly lower in the group of patients with confirmed pSS compared to those with subjective xerostomia without autoimmune background. However, only less than one-half of the measurements are below the value of 0.1 mL/min adopted as the threshold in the classification criteria at the time of diagnosis. In our study population, changes in ultrasound imaging of salivary glands occurred almost exclusively among subjects who met the SS diagnostic criteria. Another important observation was that SSA + and SSA − patients did not differ in terms of age, UWS rates, or changes in large salivary gland ultrasound scans. Therefore, LSGB should be a useful and routine component of diagnostic examinations.

SS is most likely to be suspected in patients showing signs of eye and mouth dryness. UWS rate measurements are a simple, non-invasive, and repeatable procedure providing an objective method for quantitative assessment of salivary secretion. When assessing UWS rates, possible factors responsible for xerostomia should be taken into account and eliminated already at the preliminary study preparation stage [10, 11]. When assessing the results, the patient's age and gender should also be considered since UWS rates are lower in females above 50 [4, 12]. The same holds for male patients [4, 13]. However, antihypertensive treatment was found to have no impact on UWS rates in pSS patients [4]. Despite being considered one of the major causes of decreased UWS values (an iatrogenic factor). In our analysis, we have not confirmed the relationship between the value of UWS and age. UWS values were lower in individuals over 50. The calculated Spearman's correlation coefficient was − 0.28. The *p* value for the correlation significance test was 0.1, which means that correlation exists, albeit it is very weak and not significant at the predefined significance level of 5%. Nonetheless, a correlation exists and must be examined in a larger population.

Authors of a Scandinavian study claim that lower UWS rates are negatively correlated with the immune parameters such as ANA, anti-SSA, and anti-SSB antibodies; No association between the UWS rate and the FS, patient's age, and duration of pSS could be demonstrated [14], as also confirmed by our results. We did not observe the correlation between UWS and anti-SSA positivity.

Recent studies suggested the need to revalidate UWS threshold values in the diagnostics of pSS depending on the factors mentioned above. The UWS threshold of 0.1 mL/min was proposed in 1992 when pSS classification criteria were established [2] and have not been verified ever since. Lacombe et al. [4] also observed the same results as we, that pSS patients presented higher mean UWS rates (0.3 mL/min in our study). In our group, as many as 65% of pSS patients had UWS of > 0.1 mL/min, indicating the need for a thorough evaluation of the previous UWS threshold as used in the classification criteria. Researchers of a recently published study came to similar conclusions. In a meta-analysis on UWS in SS, they suggest the need for further study to redefine UWS cut-off points to better discriminate between patients with SS vs. sicca non-SS [15].

Although LSGB is an invasive procedure, it is quite simple in technical terms and does not require significant financial resources, including anesthesiological preparation and full operating block conditions. The procedure can be performed after the patient has been prepared in the treatment room. From our experience, LSGB following topical anesthetic spray application is well-tolerated by patients and very rarely involves local complications. Patients presenting with anti-SSA antibodies were not different from antibody-negative patients in terms of age, UWS rate, or changes in major salivary gland ultrasound scans.

In addition, large-scale analyses of pSS patients revealed an association between the presence of lymphocyte infiltrations meeting the FS criteria and anti-SSA and anti-SSB antibodies, RF, higher IgG levels, and clinical signs of dryness of both mouth and eyes [16, 17].

Besides, higher FS values were associated with the possibility of lymphomas developing in the course of SS [18]. Hence, thorough histopathological evaluation of LSGB specimens, including germinal centers' assessment, may be of prognostic importance as early as at the SS diagnostic stage [19]. Given the above data, this is another reason to assess FS regardless of the value of the UWS.

The logistic regression analysis results revealed that severe US activity was an independent predictor of moderate and high pSS activity [20]. The presence of lymphoma increased significantly, with a higher risk for moderate disease activity. It is all the more indicative of the need to perform LSGBs to evaluate SS activity, particularly in patients with xerostomia and UWS of < 0.4 mL/min. In our patients, changes observable in ultrasound scans of major salivary glands were found mainly in pSS patients. They were nearly completely lacking in the control group. Given this, we believe that along with the UWS rate measurements, changes observable in ultrasound scans of major salivary glands may comprise a significant non-invasive criterion in SS diagnostics.

The limitations of this study include a small sample of the study group, which needs further studies.

In conclusion, LSGB should be performed in all suspected pSS cases, regardless of the UWS rate value. In patients with suspected pSS, only less than one-half of the UWS measurements are below the value of 0.1 mL/min adopted as the threshold in the classification criteria for pSS.
